# Chlamydiosis in Animals

**DOI:** 10.3390/ani14213130

**Published:** 2024-10-30

**Authors:** Sergio Gastón Caspe, Holly Hill

**Affiliations:** 1 Moredun Research Institute, Pentlands Science Park, Bush Loan, Penicuik EH26 0PZ, UK; holly.hill@moredun.ac.uk; 2 Animal Health Deptartment, Instituto Nacional de Tecnología Agropecuaria (INTA) EEA Mercedes, Juan Pujol al este S/N, Mercedes W3470, Corrientes, Argentina

**Keywords:** animal chlamydiosis, chlamydial zoonosis, host tropism, *Chlamydia*, psittacosis, developmental cycle

## Abstract

The *Chlamydiaceae* family consists of Gram-negative, obligate intracellular bacteria that replicate within the cells of a diverse range of hosts. These hosts include domesticated animals such as cats, dogs, and livestock, as well as wildlife like koalas and birds, exotic species such as reptiles and amphibians, and humans. Chlamydial infection can result in various clinical signs, including respiratory diseases, reproductive failures, ocular pathologies, and enteritis, though the infected organism may remain asymptomatic. In recent years, chlamydial nomenclature has undergone several revisions due to the wide range of hosts, the frequent discovery of novel strains, and the reclassification of existing ones. Given this and the clinical significance of these infections, ranging from asymptomatic to fatal, an updated review is essential. This article outlines key characteristics of *Chlamydia* species and provides an updated overview of their nomenclature, offering a concise reference for future research on chlamydial diseases.

## Simple Summary

The *Chlamydia* genus includes 17 species and 4 candidate species, comprising a group of closely related obligate intracellular bacteria that affect many animals. While each *Chlamydia* species has at least one primary host, most can infect multiple species. The clinical importance of infection varies widely, from asymptomatic cases to fatal diseases in both animals and humans. Ongoing discoveries of new species and advances in sequencing technologies continuously reshape the classification of *Chlamydia*, necessitating regular updates. This review aims to provide an accessible overview of chlamydial infections, summarising the key characteristics of *Chlamydia* species and the implications of their infections.

## 1. Introduction

The order *Chlamydiales* encompasses a group of Gram-negative bacteria that infect a wide range of hosts, including humans, domestic mammals, and wildlife [1,2]. Infections can vary from asymptomatic cases to severe conditions such as conjunctivitis, pneumonia, and reproductive disease, depending on the *Chlamydia* species and the host involved. These obligate intracellular bacteria undergo a biphasic developmental cycle, replicating within the eukaryotic host cell to form intracytoplasmic inclusions before dispersing to infect surrounding cells.

The taxonomy of *Chlamydiales* has evolved multiple times and now includes nine bacterial families: *Chlamydiaceae*, *Clavichlamydiaceae*, *Cribchlamydiaceae*, *Parachlamydiaceae*, *Parilichlamydiaceae*, *Piscichlamydiaceae*, *Rhabdochlamydiaceae*, *Simkaniaceae*, and *Waddliaceae* [1,3,4,5,6]. The family *Chlamydiaceae* includes two genera: *Chlamydia*, with 17 species (*Chlamydia abortus*, *C. avium*, *C. buteonis*, *C. caviae*, *C. crocodili*, *C. felis*, *C. gallinacea*, *C. muridarum*, *C. pecorum*, *C. pneumoniae*, *C. poikilothermis*, *C. psittaci*, *C. serpentis*, *C. suis*, *C. vaughanii*, *C. ibidis*, and *C. trachomatis*) and 3 *Candidatus* (*Ca*.) species (*Ca. C. sanzinia*, *Ca. C. corallus*, and *Ca. C. testudinis*), and *Chlamydiifrater*, which comprises 2 species (*Chlamydiifrater phoenicopteri* and *Chlamydiifrater volucris*) [3,6,7,8,9,10,11,12,13,14,15,16,17,18]. However, new species discoveries and advances in genotyping and metagenomics frequently prompt revisions in classification [6,15,19].

The dynamic nature of the *Chlamydia* genus and its ability to infect new host species complicates the study of chlamydial diseases. This review provides an updated genus overview, focusing on its taxonomy, disease associations, and historical context. It uses data from scientific articles, historical reports, and genetic databases to offer a comprehensive yet accessible perspective on animal chlamydial diseases.

For this review, databases such as PubMed, Scopus, and NCBI were used to search terms including *Chlamydia*, *Chlamydophila*, *psittacosis*, and *ornithosis*. The two authors screened the articles, selecting relevant reports or up to five references for species with multiple citations. Articles in English, German, French, and Spanish were included.

## 2. Developmental Cycle

**Figure 1:**
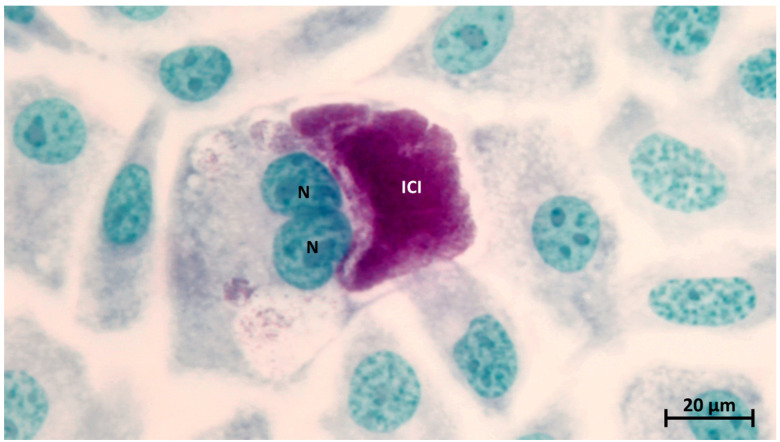
BGM cells containing intracytoplasmic chlamydial inclusion. Note the nucleus (N) surrounded by the intracytoplasmic chlamydial inclusion (ICI). Modified Zn staining.

The *Chlamydiales* cycle consists of two main morphological forms: elementary bodies (EBs), which are extracellular and infectious, measuring approximately 240–400 nm in diameter, and reticulate bodies (RBs), which are intracellular, non-infectious, but metabolically active forms ranging from 500–1600 nm in diameter (Figure 1) [20]. The cycle begins with host cell–pathogen interactions, during which EBs are internalised into eukaryotic cells through endocytosis, forming intracytoplasmic inclusions. Within these inclusions, located near the host cell nucleus, endoplasmic reticulum, and Golgi’s compartments, EBs acquire the necessary substances for replication [21,22]. Inside the inclusion, EBs transform into RBs, which then undergo consecutive rounds of intracellular replication via binary fission, utilising ATP and metabolites from the host cells until they differentiate back into the metabolically inactive infectious EBs [21,23].

**Figure 2:**
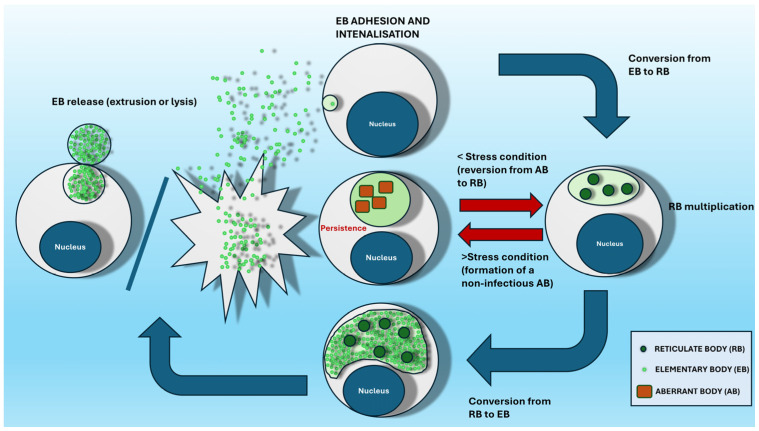
Developmental cycle.

The infectious EBs are released either through host cell lysis or extrusion of the inclusion between 48 to 72 h post-infection (depending on the species), allowing them to invade surrounding cells [24,25,26]. EBs are resistant to both physical and chemical factors in the extracellular environment and contain highly condensed nuclear material with little to no peptidoglycan in their rigid cell envelope [27]. This “spore-like” extracellular form enables EBs to survive for prolonged periods—up to months—outside their natural host [26]. Under stress conditions, such as β-lactam antibiotics, RBs can remain viable and form non-infectious aberrant bodies (ABs), which do not progress to EBs. These ABs may contribute to the persistence of the infection, leading to chronic inflammation and fibrosis [1]. This state is also reversible; if the inducing factor is removed, ABs will revert to RBs, returning to the replication phase and continuing the developmental cycle by converting back to EBs (Figure 2).

## 3. Historical Background

Chlamydial diseases, such as trachoma, have been documented since ancient times. References to granular conjunctivitis and blindness, termed “Rekha.t”, appear in Eber’s Papyrus from approximately 1550 BCE, which may be interpreted as “trachoma” [28]. The term “trachoma” itself derives from the Ancient Greek τράχωμα (trákhōma, meaning “roughness”), as reported by Plato in the *Hippocratic Corpus* in the 5th century BCE, which describes trachoma as an ocular disease. The disease became a serious health problem in Europe in the 1800s, particularly following the Napoleonic wars in Egypt, when returning troops spread it throughout large cities across the continent. Although well documented, it was not until an outbreak in Ireland in 1935 that the bacterium was described as filtrable [29,30]. In 1956, it was finally characterised by electron microscopy, and the disease was experimentally transmitted through inoculation in monkeys and human volunteers [31].

The second recognised chlamydial disease was human psittacosis, identified in the 1890s as atypical pneumonia associated with pet parrots, from which the name is derived, based on the Greek word ψιττακός (psittakós, meaning “parrot”) [32]. A global pandemic of human psittacosis occurred between 1929 and 1930 due to the shipment of exotic birds from South America to Europe and North America [33,34,35,36,37], during which *Chlamydia psittaci* was first isolated. Subsequent infections in Europe related to other bird species led to the designation “ornithosis”, derived from the Greek ornīth- (órnīs, meaning “bird”) [38,39,40,41,42,43].

The first report on ruminants of chlamydial infections was in 1909, describing transmissible vaginitis in cattle caused by “trachoma-bodies”, referenced as “*Chlamydozoa*” [44]. However, it was not until 1992 that the causal agent was determined to be *C. pecorum* [45]. In sheep, Greig first reported an abortifacient chlamydial disease in 1936 [46], later described by Stamp et al. [47] as the enzootic abortion of ewes caused by a psittacosis–LGV (lymphogranuloma venereum–trachoma) group. The accepted nomenclature of this bacterial species has undergone constant changes, evolving from *Chlamydia psittaci* immunotype-1 [48] to *Chlamydophila abortus* [49] and finally to *Chlamydia abortus* [12]. Recent studies have proposed two subtypes under this latest designation: mammalian *C. abortus* and avian *C. abortus* [15,19].

Similarly, *Chlamydia felis* was initially reported as the viral cause of pneumonia in cats and a zoonotic disease in 1942 [50] and has been referred to by several names, including psittacosis–LGV group infective agents [51], feline pneumonitis virus [52], *Bedsonia* spp. [53], feline *Chlamydia psittaci* [49], and finally, *Chlamydia felis* [12].

*Chlamydia* species are responsible for a range of infections, including sexually transmitted infections (STIs), respiratory infections, and ocular diseases. Understanding the taxonomy of the *Chlamydia* genus bacteria is crucial for studying the biology, epidemiology, and pathology and developing effective diagnostic and therapeutic strategies.

In 1971, the order *Chlamydiales*, initially comprised solely of the *Chlamydia* genus, was established based on characteristics such as susceptibility to sulfonamide and the presence of glycogen inclusion bodies detectable by iodine staining. Changes to the classification criteria to include serological and molecular evaluations led to the order’s expansion to include *Chlamydia trachomatis* and *Chlamydia psittaci* through serotyping [54], as well as *C. pneumoniae* [55] and *C. pecorum* [45,56] via DNA analysis. After 1999, taxonomy relied on phylogenetic analysis of the 16S and 23S RNA genes, resulting in the differentiation of five additional species: *C. caviae*, *C. felis*, *C. muridarum*, *C. pneumoniae*, and *C. suis*. As whole-genome sequencing techniques became widely available, more in-depth analyses were possible. This method remains the current standard for classifying new chlamydial species [49]. To date, there are a total of 17 recognised species and 3 candidatus species in the order.

## 4. Genomic and Phylogenetic Analysis

The first genome sequence of *Chlamydia trachomatis* strain D/UW-3/CX was published in 1998 [57], providing foundational insights into the genetic makeup of *Chlamydia* and paving the way for further research and advancements in the field. Since then, next-generation sequencing technologies, including whole-genome sequencing, have vastly expanded the number of publicly available *Chlamydia* genomes. These technological improvements have greatly enhanced our understanding of *Chlamydia*’s genetic diversity and evolutionary dynamics, facilitating the rapid identification of new and potential members of the genus [19,58]. Over the past decade, this progress has led to the recognition of six new *Chlamydia* species: *C. buteonis* [9] and *C. gallinacea* [59] in birds; *C. crocodili* [19], *C. poikilothermis* [60], and *C. serpentis* [60] in reptiles; and *C. vaughanii* [18] in fish. Additionally, three candidatus species have been proposed in reptiles: *Ca*. *Chlamydia sanzinia* [61], *Ca*. *Chlamydia testudinis* [62], and *Ca*. *Chlamydia corallus* [63]. Two novel species in birds have also been classified under the newly established *Chlamydiifrater* genus: *C. phoenicopterid* and *C. volucris* [7].

A sequence analysis of the chlamydial genome revealed it to be relatively small and well-conserved, ranging from 1 to 1.2 million base pairs (Mbp). It is organised as a single circular chromosome, typically between 900 and 1500 coding sequences (CDSs), depending on the species [64,65,66]. This compact genome results from genetic streamlining rather than degradation, with genetic loss attributed to the bacteria’s adaptation to an intracellular lifestyle [67]. The level of genetic diversity due to single-nucleotide polymorphisms (SNPs) varies between species and can be linked to differences in pathogenic properties, tissue tropism, and host tropism [19,60,62,68].

Most species, apart from mammalian *C. abortus*, human *C. pneumoniae*, and rarely *C. trachomatis* strains, contain a plasmid of approximately 7.5 kbp [69]. This plasmid is highly conserved and contains eight genes (Pgp 1–8) involved in plasmid maintenance (Pgp 1, 2, and 6) and replication (Pgp 7 and 8), as well as RNA of unknown functionality (Pgp 4) [69,70]. Pgp 3 and 5 may contribute to increased virulence and plasmid-dependant pathogenicity in some strains [71,72]. While plasmids are generally known to carry antibiotic-resistance genes, this seems to be lacking in chlamydial plasmids [67]. Instead, tetracyclin resistance has been detected in *C. suis* associated with tet(C) genomic islands within the chromosome, likely a result of horizontal gene transfer [73]. The mobilizable plasmid pRAS3.2 from *Aeromonas salmonicida* exhibits a high level of nucleotide identity (99.9%) and a conserved gene sequence with the Tet-island, including the tet operon with tetA(C) and its repressor-encoding tetR(C), suggesting that it may have been acquired through coinfection via pigs ingesting infected fish meal [74]. Recombination is believed to play a key role in the transmission of the tetA(C) gene between circulating *C. suis* strains [75].

**Figure 3:**
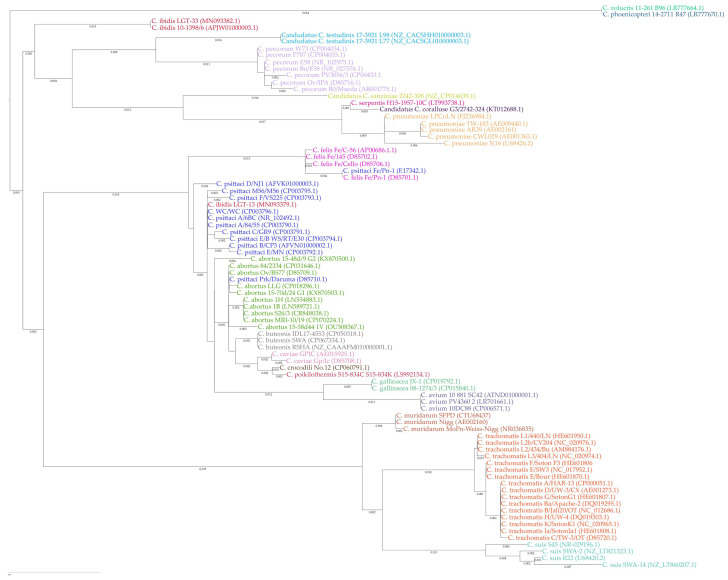
Phylogenetic analysis of the *Chlamydia* genus. Phylogenetic tree of 16S rRNA sequences of representative strains of *Chlamydia* spp. Phylogeny is based on 1356 bp MAFT alignment; the tree was constructed using the Maximum Likelihood method RAxML combined with 1000 bootstrap replicates as implemented in MegaAlign Pro 17, coloured by species.

Phylogenetic characterisation of the *Chlamydia* genus has traditionally relied on 16S rRNA sequence analysis (shown in Figure 3), revealing that members of the genus share over 95% identity, with a within-species homology of 90% [49,68]. Recently, following the recommendations of the International Committee on Systematics of Prokaryotes Subcommittee on the taxonomy of Chlamydiae [6], a multilocus sequence typing scheme (MLST) was proposed by Pillonel et al. [76]. MLST classifies isolates based on up to 20 taxonomically reliable and informative markers, which enhances resolution sensitivity and phylogenetic robustness.

## 5. Host Tropism

Until 2021, when the genus *Chlamydiifrater* was identified in flamingos [7], the *Chlamydiaceae* family consisted solely of the *Chlamydia* genus. *Chlamydia* species affect a broad range of animals, including humans, causing a variety of diseases [1,2,3,7,8,10,14,77,78]. While *C. trachomatis* is a leading cause of bacterial sexually transmitted diseases in humans, other species, such as *C. abortus*, *C. pneumoniae*, *C. felis*, *C. suis*, and *C. psittaci*, are also significant zoonotic pathogens [4,79,80,81,82,83,84,85]. Although *Chlamydia* species can infect nearly all animals, each species typically has a primary host where the infection is endemic (Table 1).

### 5.1. Avian Chlamydiosis

Traditionally, avian chlamydiosis referred to the disease caused by *C. psittaci*. However, additional novel avian species, *C. gallinacea*, *C. avium* [65], *C. buteonis* [8], *C. ibidis* [10], *C. phoenicopterid*, and *C. volucris* [7], are now classified under this term.

#### 5.1.1. *C. psittaci*

*C. psittaci* infects over 465 domestic and wild bird species, with those from the order Psittaciformes being the most commonly affected, hence the term “psittacosis” [1]. In non-psittacine birds, the disease is known as “ornithosis”, though the disease is essentially the same in all avian species [2]. Clinical signs include fever, rash, inappetence, muscle aches, fatigue, respiratory distress with cough, and, less frequently, severe pneumonia [80,118,135,136]. While birds are the natural host, *C. psittaci* can also infect various mammals (cattle, goats, sheep, horses, pigs, foxes, and dogs), including humans [90,137,138,139,140,141]. Human psittacosis is mainly an occupational hazard for those working with birds, such as those responsible for their care, veterinarians, and slaughter and processing plant workers [23,84,90,142]. In humans, the disease ranges from mild febrile illness to severe pneumonia, with potential complications like myocarditis, hepatitis, and meningoencephalitis that progress into lethal systemic disease [143,144,145].

#### 5.1.2. *C. gallinacea*

*C. gallinacea* primarily affects domestic poultry (chicken, guinea fowl, and turkeys), leading to a reduced weight gain [100,138], and has been linked to cases of atypical pneumonia in European poultry slaughterhouse workers and backyard poultry farmers [13,65,98,135,138,146,147]. It also infects wild birds (psittacine and woodcocks) and other mammals [99,104,146,148,149].

#### 5.1.3. *C. avium*

*C. avium* has been found in different species of pigeons and psittacines [65,147,150,151,152]. Typically, a *C. avium* infection is asymptomatic, though it can cause respiratory disease in pigeons, catarrhal enteritis, and splenomegaly in psittacines [65,138,153,154]. No human cases have been documented, and its zoonotic potential remains unclear.

#### 5.1.4. *C. buteonis*

*C. buteonis* was isolated from red-shouldered hawks (*Buteo jamaicensis)* and Swainson’s hawks (*Buteo swainsonii)*, which exhibited ocular signs, and from asymptomatic gyrfalcons (*Falco rusticolus*) [8,91,92]. Necropsies revealed severe ocular lesions (ulcerative conjunctivitis) and mild lesions such as non-suppurative moderate pneumonia, hepatitis, and splenitis [3]. No zoonotic cases have been reported.

#### 5.1.5. *C. ibidis*

*C. ibidis* was first isolated from cloacal swabs and faecal samples collected from asymptomatic *feral African Sacred Ibises* (*Threskiornis aethiopicus*) during officially sanctioned population control in France. *C. ibidis* has also been identified in faecal samples from Crested Ibises (*Nipponia nippon)* in China [10,155]. No clinical signs or zoonotic infections have been reported.

### 5.2. Chlamydiosis in Reptiles

Three species of *Chlamydia* (*C. serpentis*, *C. crocodile*, and *C. poikilothermis*) and three candidate species (*C. sanzinia*, *C. corallus*, and *C. testudines*) are known to infect reptiles [3,14,78,117,133]. *C. serpentis* and *C. poikilothermis* have been isolated from asymptomatic snakes, including corn snakes (*Pantheropis guttatus)* and the green bush viper (*Atheris squamigera)*, respectively. *C. crocodili* was found in an asymptomatic captive Siamese crocodile (*Crocodylus siamensis*) [18,78,117]. The candidate species *C. sanzinia* and *C. corallus* were found in captive asymptomatic boas, specifically the Madagascar Tree boa (Sanzinia madagascariensis volontany) and the Amazon Basin Emerald Tree boa (*Corallus batesii*) [113]. In contrast, *C. testudinis* was isolated from Spur-Thighed tortoises (*Testudo graeca*), showing ocular clinical signs [3,133].

### 5.3. Chlamydiosis in Fish

*Chlamydia*-related species (species from the families *Simkaniaceae*, *Parilichlamydiaceae*, *Piscichlamydiaceae*, and *Clavochlamydiaceae*) have long been linked to epitheliocystis in fish [156]. However, it was not until 2023 that a *Chlamydia* species, *C. vaughanii*, was proposed after it was isolated from dead bushy-mouth catfish (*Ancistrus dolichopterus*) in a private aquarium [17]. No clinical lesions in fish or zoonotic relevance have been reported.

### 5.4. Chlamydiosis in Mammals

#### 5.4.1. *C. abortus*

Originally, *C. abortus* was identified in mammals as the etiological agent of the enzootic abortion of ewes (EAE). However, whole-genome sequencing and comparative genomic analyses of previously classified atypical *C. psittaci* strains led to their reclassification as avian *C. abortus* [15,19]. Consequently, *C. abortus* now encompasses two subgroups: mammalian and avian [6]. Avian strains have been found in birds like waterfowl, corvids, and parrots and possess a plasmid and cytotoxin gene, distinguishing them from mammalian strains [15,59,157]. While mammalian *C. abortus* is zoonotic, the zoonotic potential of avian *C. abortus* remains unclear.

**Figure 4:**
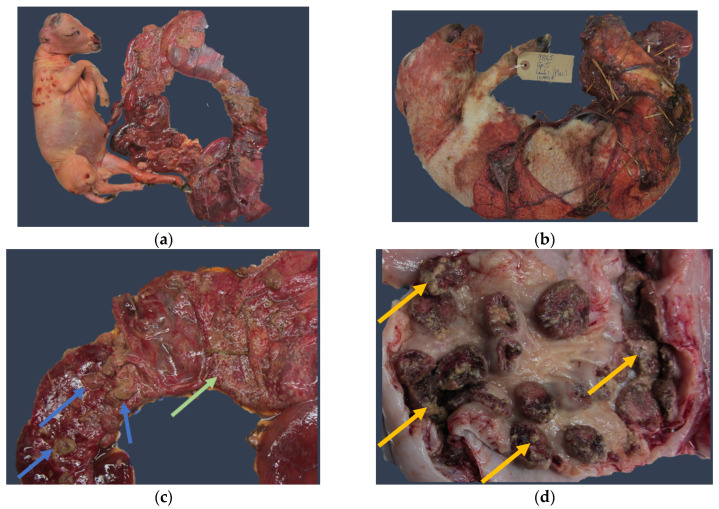
Chlamydiosis in sheep: (**a**) aborted foetus and its corresponding necrotic placenta in the last period of gestation; (**b**) foetus aborted on-term; (**c**) placenta showing subepithelial oedema, necrosis of trophoblast in the inter-cotyledonary area (green arrow), and cotyledons (blue arrows); (**d**) uterine caruncles showing tissular necrosis as greyish debris (yellow arrows).

Mammalian *C. abortus* primarily affects small ruminants but can infect humans, causing acute atypical pneumonia, abortion, and pelvic inflammatory disease, often accompanied by unusually heavy vaginal discharge that may lead to adverse pregnancy outcomes [26,82,158,159,160,161,162]. In sheep, it typically causes late-term abortions (2–3 weeks before expected lambing), stillbirths, or neonatal deaths (weak lambs that fail to survive beyond 48 h) [2,16,89]. Typical lesions associated with abortion are suppurative necrotising placentitis and endometritis (Figure 4). Transmission occurs through contact with infected placentae, genital fluid discharge following abortion/lambing, or the coats of foetuses or stillbirth animals, which can contaminate the environment [2,163]. Though primarily reproductive, infection can also sporadically cause bronchiolitis, interstitial pneumonia, mastitis, and periportal focal hepatitis [164].

Disease control in flocks is currently achieved using commercial vaccines, including inactivated (Mydiavac, Novartis Animal Health, London, UK; Chlamysure, Onderstepoort Biological Products, Pretoria, South Africa; INMEVA, Laboratorios Hipra, S.A, Giron, Spain) and live vaccines containing the attenuated temperature-sensitive *C. abortus* 1B strain (Enzovax^®^, MSD Animal Health (Rahway, NJ, USA); CEVA *Chlamydia*^®^, CEVA Animal Health (Paris, France)), which are widely used across many countries [165,166]. Inactive vaccines are generally considered safer than live vaccines but may alter the antigenicity of surface proteins. In contrast, live vaccines preserve the antigenic structures of the surface membrane and proteins, which could be important for the protective immune response. However, recent studies have suggested that the 1B strain in live vaccines might contribute to disease in some cases [87,166,167,168,169]. Subcellular vaccines, such as those based on the chlamydial outer membrane complex (COMC) and octyl glucoside (OG)-COMC), show promise as an alternative. These vaccines elicit a protective immune response comparable to live vaccines without the ability to replicate or cause infection in animals [170].

#### 5.4.2. *C. caviae*

The primary host of *C. caviae* is the guinea pig. Clinical signs range from asymptomatic to severe suppurative conjunctivitis, conjunctival chemosis, follicular hypertrophy, and pannus [93,95,171,172]. Venereal infection can also affect the urogenital tract, causing ascending urethral infection, cystitis, endometritis, and salpingitis. Vertical transmission can result in neonatal conjunctivitis in pups [173,174,175,176,177,178,179].

*C. caviae* infections have occasionally been detected in other animals like cats, dogs, rabbits, and horses and may be associated with conjunctivitis and respiratory disease [180,181]. Human cases linked to direct exposure to infected guinea pigs typically involve conjunctivitis and occasionally severe respiratory disease [95,171,176].

#### 5.4.3. *C. felis*

In cats, *C. felis* causes conjunctivitis and mild respiratory distress [97,182]. Ocular diseases progress from mild, unilateral conjunctivitis to more severe bilateral conjunctivitis, marked by chemosis, hyperaemia protrusion of the nictitating membrane, blepharospasm, and mucopurulent discharge [183,184]. While spontaneous recovery has been reported, untreated cases often lead to chronic conjunctivitis. Ocular shedding can persist for up to eight months, allowing recovered cats to serve as asymptomatic carriers [183]. Transmission occurs through aerosols, with infected cats—whether symptomatic or not—spreading the pathogen to healthy cats in close contact [121,183,184,185]. Experimental inoculation has also produced clinical signs such as lameness, peritonitis, gastritis, and vaginal discharge [183,186].

The first zoonotic case of feline chlamydia was associated with atypical human pneumonia [50]. Most human cases of acute follicular keratoconjunctivitis are linked to pet owners whose cats recently experienced rhinitis and conjunctivitis [83,94].

#### 5.4.4. *C. muridarum*

*C. muridarum* naturally infects members of the Muridae family, such as mice and hamsters, though it can occasionally infect other species, including chickens [100,187]. First isolated in 1942 from the lungs of asymptomatic albino Swiss mice [188], *C. muridarum* is associated with bronchopneumonia in mice and ileitis in hamsters [189,190,191]. Experimental genital tract infections have demonstrated their potential to cause venereal disease [103,192].

Unlike *C. trachomatis*, *C. muridarum* lacks a tryptophan operon, causing a distinct response to *IFN-γ*. Nevertheless, due to its ability to be transmitted genitally, it exhibits a similar pathological profile to human chlamydiosis, making it a widely used experimental model for studying *C. trachomatis*. In female mice, experimental genital infections result in oviduct occlusions and hydrosalpinx [187,193], while in males, urethritis occurs without affecting fertility or sperm quality [194].

#### 5.4.5. *C. pecorum*

*C. pecorum* is responsible for a wide range of diseases in small and large ruminants, including conjunctivitis, encephalomyelitis, enteritis, infertility, vaginitis, endometritis, abortion, enteritis, mastitis, polyarthritis, and pneumonia [1,105,108,195,196,197,198,199]. Its zoonotic potential remains unknown.

In Australian wildlife, *C. pecorum* has notably affected koalas, causing conditions such as mucopurulent keratoconjunctivitis, blindness, infertility, cystic dilatation, bursitis of the ovarian bursa, endometritis, vaginitis, pyometra, cystitis, hydronephrosis, renal fibrosis, epididymitis, and prostatitis [1,106,108,109,200]. While venereal transmission is the primary route of infection in koalas, oral–faecal transmission may also occur due to their coprophagic behaviour [201].

#### 5.4.6. *C. suis*

*C. suis* shares biological similarities with *Chlamydia trachomatis*, leading to its early description as “*C. trachomatis* infection in pigs” [202,203,204,205]. While often associated with subclinical infections in pigs, *C. suis* can also cause bronchopneumonia, keratoconjunctivitis, polyserositis, pericarditis, arthritis, mastitis, metritis, agalactia, abortions, and enteritis, particularly in piglets [202,203,206,207,208,209]. The overuse of antibiotics in pig production has contributed to antimicrobial resistance (AMR) in this species [73,210,211,212,213]. Zoonotic transmission of C. suis has been reported in pig slaughterhouse workers and farmers, presenting as conjunctivitis [127,129,214,215].

### 5.5. Human Chlamydiosis

Human chlamydial infections are caused by *C. trachomatis* and *C. pneumoniae*, leading to distinct diseases. *C. trachomatis* is associated with eye and urogenital infections, often resulting in blindness and sexually transmitted infertility, while *C. pneumoniae* is responsible for respiratory infections linked to bronchitis, pneumonia, coronary disease, arthritis, and asthma [110,112,115,216,217,218,219].

#### 5.5.1. *C. trachomatis*

Trachoma has been recognised for centuries as the most common cause of sexually transmitted diseases worldwide [28]. Humans are the only known host, with no evidence of transmission to animals. Although many infections are asymptomatic, complications can arise. Ocular infections may progress to conjunctivitis, potentially leading to blindness. In men, *C. trachomatis* can cause seminal vesiculitis, epididymitis, urethritis, and infertility. In women, it can cause endometritis with abnormal vaginal discharges or bleeding and urethritis with dysuria [25,220,221]. Recent studies have suggested an association between *C. trachomatis* infection and cervical cancer [123]. The overuse of antibiotics used to control the infection has contributed to the development of AMR and subsequent treatment failures [212,221,222,223].

#### 5.5.2. *C. pneumoniae*

Infections with *C. pneumoniae* typically cause respiratory infections in humans and have been associated with cardiocirculatory pathologies and atherosclerosis in respiratory diseases [112,224].

Although humans are the main host, infections have been documented in other species, including horses, koalas, frogs, and reptiles. In horses, *C. pneumoniae* may lead to subclinical infections or conjunctivitis, while in marsupials, it can cause corneal opacity and blepharitis [1,106,109]. In reptiles and amphibians, infections have been linked to severe systemic granulomatous inflammatory diseases, such as necrotising enteritis, myocarditis, and hepatitis [106,111,113,114,116,225].

### 5.6. Chlamydial Zoonosis

**Figure 5:**
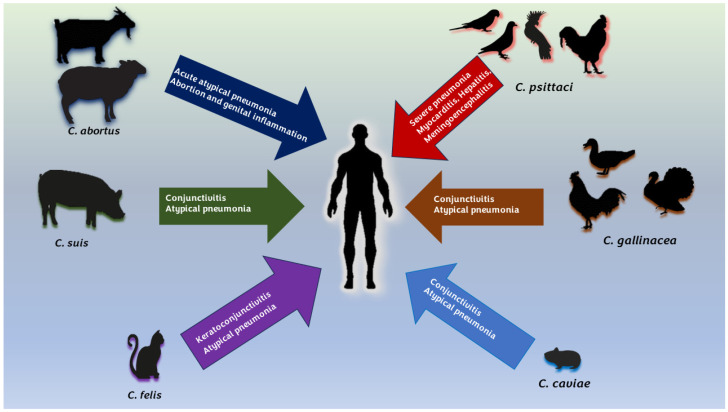
Chlamydial zoonotic agents and their main hosts. Arrow contains the main diseases related to human infections.

While *C. psittaci* and *C. abortus* are as well-established zoonotic pathogens [1,2,26,82,143,145,158,226], other species, such as *C. felis*, *C. caviae*, *C. gallinacean*, and *C. suis*, have also been implicated as possible zoonotic agents capable of causing respiratory and ocular infections in humans [83,127,171,214] (Figure 5).

Human infections with *C. psittaci* have been widely reported, typically causing respiratory infections characterised by fever, chills, headache, myalgia, nonproductive cough, and respiratory distress. However, atypical symptoms, including erythroderma, dermatomyositis, myocarditis, encephalitis, hepatitis, keratoconjunctivitis, ocular lymphomas, acute respiratory distress syndrome, and multiple organ failure, have also been observed [84,227]. Human infections tend to occur in people with occupational exposure, particularly avian farmers, slaughterhouse workers, veterinarians, and pet shop employees.

*Chlamydia abortus* infections have been associated with pregnant farmers in close contact with small ruminants. Clinical symptoms include fever, headache, dry cough, septic infarction shock, severe thrombocytopenia, and abortions [81,82,158,159,160,161,162,228]. Lesions found in human placentas closely resemble those observed in typical ovine chlamydiosis, specifically suppurative necrotising placentitis [81,158,162].

Other zoonotic chlamydial species, such as *C. felis*, *C. caviae*, *C. gallinacean*, and *C. suis*, generally cause conjunctivitis and atypical pneumonia in humans. *C. felis* infections have been reported in cat owners exposed to cats with conjunctivitis and rhinitis [83,185,229,230] and in rare cases have been linked to glomerulonephritis and endocarditis [231]. Infections with *C. caviae* have been found in pet owners, although no clinical signs were observed in their pets [95,171]. Additionally, *C. gallinacea* and *C. suis* have caused infections in farmers and slaughterhouse employees [90,98,127,129,135,149,214].

## 6. One Health Perspective

One Health is a cohesive strategy aimed at achieving a sustainable balance and improving the well-being of humans, animals, and ecosystems through integration. It has gained prominence, particularly concerning emerging infectious and zoonotic diseases transmitted between animals and humans. Human activities such as urban expansion and deforestation have significantly altered natural ecosystems, creating new opportunities for transmission. For instance, deforestation of the Amazon, where livestock farms are replacing virgin native rainforests, has led to encroachment on wildlife areas, alterations in their routes, accessibility to peripheral regions and, principally, environmental contamination [147,232,233]. This disruption in migration patterns and habitats of various species creates a novel interaction between domestic and wildlife species that may carry diseases unfamiliar to one another.

*Chlamydia* spp. are no exception to this trend. Many new *Chlamydia* species and interspecies transmission instances have been discovered recently. While *Chlamydia* bacteria are associated with specific animal species, they exhibit a remarkable capacity to infect others. Numerous examples of this have been reported, including infections of koalas with *C. pecorum*, reptiles with *C. pneumoneae*, horses with *C. psittaci*, and humans with *C. psittaci*, *C. gallinacea*, *C. felis*, *C. suis*, *C. abortus*, and *C. caviae*. These infections are often associated with non-native animal species, occupational exposure, or the ownership of exotic pets. Environments like zoos, pet markets, and food markets—where animals from different ecosystems are kept in close proximity—further facilitate the spread of these pathogens. Additionally, the global commercialisation of wildlife and improved connectivity have accelerated the potential risk of disease dissemination.

It is essential to recognise that animal chlamydiosis ranges from asymptomatic infection to severe diseases, making detection difficult. Asymptomatic animals in endemic areas can transmit infections to other species, leading to serious or fatal illnesses. The discovery of new *Chlamydia* species and the expansion of host ranges may introduce new zoonotic risks. For example, the identification of *C. abortus* in bird species (avian *C. abortus*) raises concerns about its potential evolution in new epidemiological contexts. Furthermore, the ability of chlamydial species to cross host barriers highlights the need for improved diagnostic tools, surveillance, and control measures.

Active surveillance of chlamydial infections in animals and humans might be the key to environmental sustainability. By adopting a One Health approach, researchers, healthcare professionals, and policymakers can develop cross-disciplinary strategies to address emerging and resurging diseases, with wildlife health as an essential component in global disease prevention. This collaborative approach is vital for building a more resilient and sustainable global health system.

## 7. Conclusions

The *Chlamydiaceae* family consists of Gram-negative bacteria of significant importance to human and animal health worldwide, contributing to considerable economic losses in livestock production. Ongoing research is crucial for identifying new strains and hosts and understanding their epidemiological impact on domestic animals and wildlife. Given their zoonotic potential, deepening our understanding of their potential effects on human health is critical. This review provides an updated overview of the latest knowledge surrounding these bacteria.

## Figures and Tables

**Table 1:** Members of the *Chlamydiaceae* family, the main hosts, and the diseases caused.

Genus	Species	Primary Host	Typical Disease	Secondary Host	Disease in Secondary Host	References
*Chlamydia*	*C. abortus*	Small ruminants and camelids	Late-gestation abortion/stillbirth	Humans, horses, cattle, and fur animals	Abortion and atypical pneumonia	[79,86,87,88,89]
	*C. avium*	Multiple bird species	Respiratory symptoms	Unknown	Unknown	[59,61,65,90]
	*C. buteonis*	Raptor birds	Unknown	Unknown	Unknown	[8,91,92]
	*C. caviae*	Guinea pigs	Conjunctivitis and urogenital infections	Humans and other mammals	Conjunctivitis and atypical pneumonia	[93,94,95]
	*C. crocodili*	Crocodile	Asymptomatic	Unknown	Unknown	[18]
	*C. felis*	Felines	Conjunctivitis, pneumonia, and urogenital infections	Humans and dogs	Conjunctivitis and atypical pneumonia	[83,96,97]
	*C. gallinacea*	Domestic poultry	Lower weight gain	Humans	Conjunctivitis and atypical pneumonia	[20,98,99,100,101]
	*C. Ibidis*	Ibides	Unknown	Unknown	Unknown	[10]
	*C. muridarum*	Rodents	Urogenital disease	Unknown	Unknown	[94,102,103]
	*C. pecorum*	Ruminants	Urogenital disease, reproductive failures, sporadic encephalitis, and enteritis	Koala	Conjunctivitis, blindness, pneumonia, and urogenital diseases	[104,105,106,107,108,109]
	*C. pneumoniae*	Humans	Respiratory diseases, arthritis, neoplasia, and coronary disease	Amphibians and reptiles	Necrotising enteritis, myocarditis and hepatitis	[110,111,112,113,114,115,116]
	*C. poikilothermis*	Snakes	Asymptomatic	Unknown	Unknown	[78,117]
	*C. psittaci*	Psittacines	Respiratory diseases	Humans, birds, and mammals	Respiratory symptoms, myalgia, abortion, and placentitis (equines)	[61,80,83,90,118,119,120,121,122]
	*C. trachomatis*	Humans	Reproductive symptoms, conjunctivitis, and blindness			[63,94,123,124,125]
	*C. serpentis*	Snakes		Unknown	Unknown	[3,78]
	*C. suis*	Swine	Respiratory disorders, conjunctivitis, enteritis, and reproductive failure	Humans	Conjunctivitis and atypical pneumonia	[126,127,128,129,130,131,132]
	*C. vaughanii*	Fishes	Epitheliocystis	Unknown	Unknown	[17]
	*Ca. Chlamydia sanzinia*	Turtles and tortoises	Unknown	Unknown	Unknown	[133]
	*Ca. Chlamydia testudinis*	Tortoise	Conjunctivitis	Unknown	Unknown	[134]
	*Ca. Chlamydia corallus*	Snakes	Unknown	Unknown	Unknown	[77]
*Chlamydiifrater*	*C. phoenicopteri*	Flamingo	Unknown	Unknown	Unknown	[7]
	*C. volucris*	Flamingo	Unknown	Unknown	Unknown	[7]

## Data Availability

Not applicable.
